# Sequencing of Immunotherapy and Outcomes in Operable Clinical Stage III Melanoma: A National Cohort Study

**DOI:** 10.1002/jso.27933

**Published:** 2024-10-03

**Authors:** Anushka Dheer, Gabriella N. Tortorello, Neha Shafique, Mohammad S. Farooq, Tara C. Mitchell, Xiaowei Xu, John T. Miura, Giorgos C. Karakousis

**Affiliations:** ^1^ Department of Surgery Hospital of the University of Pennsylvania Philadelphia Pennsylvania USA; ^2^ Division of Hematology/Oncology Hospital of the University of Pennsylvania Philadelphia Pennsylvania USA; ^3^ Division of Pathology and Laboratory Medicine Hospital of the University of Pennsylvania Philadelphia Pennsylvania USA

**Keywords:** immunotherapy, melanoma, neoadjuvant immunotherapy, stage III melanoma

## Abstract

**Background and Objectives:**

The impact of neoadjuvant immunotherapy (NIT) on overall survival (OS) in patients with resectable stage III melanoma remains unknown. We sought to identify factors associated with receipt of NIT and survival outcomes in patients with clinical stage III melanoma undergoing surgery.

**Methods:**

The National Cancer Database (2016–2020) was used to identify patients with clinical stage III melanoma who underwent surgery and received either NIT or adjuvant immunotherapy (AIT) only. Multivariable regression, Kaplan–Meier, and Cox proportional hazard methods were used to analyze variables of interest.

**Results:**

Patients with clinical N3 disease had 2.5 times the odds of NIT compared to those with N1 disease (95% CI 1.74–3.49). There was no difference in 3‐year OS between the two cohorts: 79% (95% CI 73%–85%) for NIT patients and 75% (95% CI 73%–76%) for AIT patients (*p* = 0.078). Patients with N2/N3 disease had improved 3‐year OS of 79% with NIT versus 71% for AIT‐only (HR 0.61, 95% CI 0.38–0.97, *p* = 0.037).

**Conclusions:**

NIT is given more selectively to clinical stage III patients with more advanced N category disease. Despite significant differences in N category between groups, there was no difference in OS observed at 3 years, and NIT was associated with a survival advantage among N2/N3 patients.

## 1 Introduction

There has been growing interest in neoadjuvant approaches in the management of oligometastatic, resectable melanoma, particularly with the introduction of immune checkpoint blockade agents [1, 2, 3, 4, 5, 6, 7, 8, 9, 10, 11]. Neoadjuvant immunotherapy therapy (NIT) may offer both therapeutic and prognostic benefit [12, 13, 14]. First, NIT may pathologically downstage tumors, allowing for a decrease in the extent of surgical resection—potentially with fewer operative complications—as demonstrated in the PRADO trial [15]. Furthermore, evaluating pathological response to NIT can provide insight into prognosis and tumor biology, which may frame subsequent treatment decisions [15]. Moreover, administration of immunotherapy before surgery may stimulate a more robust innate and adaptive immune response by exposing the immune system to a higher concentration of tumor antigen [11, 16, 17].

A recent randomized phase 2 clinical trial (SWOG 1801) of patients diagnosed with predominantly resectable clinical stage III melanoma demonstrated an improved 2‐year event‐free survival of 23%, favoring a neoadjuvant/adjuvant approach with a PD‐1 inhibitor compared to adjuvant PD‐1 therapy alone [17]. Even more recently, the phase 3 NADINA trial further demonstrated improved event‐free‐survival for resectable, macroscopic stage III melanoma patients randomized to NIT plus response‐driven adjuvant immunotherapy (AIT) compared to patients undergoing surgery first followed by AIT [18]. Despite the promise of neoadjuvant immune checkpoint blockade, the impact of NIT on overall survival (OS) in patients with melanoma remains unknown. In this study, we examined a large national cohort to identify factors associated with receipt of NIT and survival outcomes in patients with clinical stage III melanoma undergoing surgery.

## 2 Materials and Methods

The National Cancer Database (NCDB) was used to identify patients with AJCC clinical stage III melanoma (N1‐N3, M0) diagnosed between 2016 and 2020 who underwent definitive surgical resection of their primary tumor as well as either NIT or AIT‐only. This database, which comprises more than 1500 Commission on Cancer‐accredited healthcare centers, captures more than two‐thirds of new cancer diagnoses made nationally on an annual basis [19]. Patients who received perioperative chemotherapy or BRAF/MEK targeted therapy were excluded. Patients with missing data pertaining to their treatment sequence, follow‐up time, or vital status were also excluded. Multivariable logistic regression modeling was used to identify predictors of NIT use. OS from the day of diagnosis was determined using the Kaplan–Meier method and Cox proportional hazards modeling. All analyses were performed using the R project for statistical computing.

## 3 Results

Of 2771 patients identified, 2526 (91%) received AIT‐only and 245 (9%) received NIT. The median age was 63 (interquartile range [IQR] 51–72), 96% of patients were White, and 66% were male (Table 1). Factors associated with receipt of NIT included being treated at an academic center (60% vs. 46%, *p* < 0.001) and clinical N category (7.6% of N1, 8.2% of N2, and 17% of N3 patients, *p* < 0.001). Accordingly, the NIT group had a greater proportion of patients with N2/N3 clinical nodal disease, with 45% of patients presenting with N2/N3 disease (24% N2, 21% N3) compared to the AIT‐only group, in which 36% of patients had N2/N3 clinical nodal disease (26% N2, 10% N3) (*p* < 0.001). There was no significant difference in patient age, sex, race, Charlson–Deyo combined comorbidity (CDCC) score, or insurance status between the two groups. On multivariable logistic regression, these same factors were associated with the receipt of NIT. Patients with clinical N3 disease had 2.5 times the odds of NIT compared to those with N1 disease (OR 2.5, 95% confidence interval [CI] 1.74–3.49, *p* < 0.001). Patients treated at an academic center had 1.8 times the odds of NIT compared to those treated elsewhere (OR 1.8, 95% CI 1.4–2.4, *p* < 0.001). The median number of total nodes examined was 14 for NIT patients compared to 6 in the AIT‐only group (*p* < 0.001), with a median of 1 positive node for both groups and an average of 4 positive nodes for the NIT cohort compared to 3 for AIT‐only. The median time to definitive surgical resection for the AIT‐only group was 33 days (IQR 19–49), while the median time for the NIT group was 127 days (IQR 96–175, *p* < 0.001).

**Table 1:** Characteristics of the patient cohort and univariate analysis by the timing of immunotherapy.

	Overall (*N* = 2771)	Adjuvant‐only (*N* = 2526)	Neoadjuvant (*N* = 245)	*p*‐value
Age, median (IQR)	63 (51, 72)	63 (51, 72)	63 (52, 73)	0.5
Sex				0.5
M	1816 (66%)	1651 (65%)	165 (67%)	
F	955 (34%)	875 (35%)	80 (33%)	
Race				0.11
White	2672 (96%)	2441 (97%)	231 (94%)	
AAPI	49 (1.8%)	40 (1.6%)	9 (3.7%)	
Black	30 (1.1%)	27 (1.1%)	3 (1.2%)	
Other/unknown	20 (0.7%)	18 (0.7%)	2 (0.8%)	
Ethnicity				0.053
Non‐Hispanic	2631 (95%)	2404 (95%)	227 (93%)	
Hispanic	37 (1.3%)	35 (1.4%)	2 (0.8%)	
Other/unknown	103 (3.7%)	87 (3.4%)	16 (6.5%)	
Insurance				> 0.9
Private	1277 (46%)	1158 (46%)	119 (49%)	
Medicare	1174 (42%)	1073 (42%)	101 (41%)	
Medicaid	182 (6.6%)	169 (6.7%)	13 (5.3%)	
Uninsured	74 (2.7%)	67 (2.7%)	7 (2.9%)	
Hospital status				< 0.001*
Academic	1298 (47%)	1150 (46%)	148 (60%)	
Community	768 (28%)	724 (29%)	44 (18%)	
Integrated network	407 (15%)	374 (15%)	33 (13%)	
Other or unknown	298 (11%)	278 (11%)	20 (8.2%)	
CDCC score				0.2
0	2211 (80%)	2020 (80%)	191 (78%)	
1	366 (13%)	325 (13%)	41 (17%)	
2	101 (3.6%)	96 (3.8%)	5 (2.0%)	
3	93 (3.4%)	85 (3.4%)	8 (3.3%)	
Clinical N category				< 0.001*
1	1739 (63%)	1606 (64%)	133 (54%)	
2	727 (26%)	667 (26%)	60 (24%)	
3	305 (11%)	253 (10%)	52 (21%)	
Nodes examined, median (IQR)	7 (2, 21)	6 (2, 21)	14 (3, 27)	< 0.001*
Positive nodes				0.7
Median (IQR)	1 (1, 3)	1 (1, 3)	1 (1, 4)	
Mean	2.83	2.71	4.13	
Ulceration				0.006*
Present	1494 (54%)	1368 (54%)	126 (51%)	
Not present	1051 (38%)	965 (38%)	86 (35%)	
Unknown	226 (8.2%)	193 (7.6%)	33 (13%)	
Breslow depth, median (IQR)	4.0 (2.1, 7.0)	4.0 (2.1, 7.0)	4.1 (2.1, 7.0)	> 0.9
Primary site				0.2
Extremities	1115 (40%)	1012 (40%)	103 (42%)	
Trunk	863 (31%)	794 (31%)	69 (28%)	
Head/neck	692 (25%)	633 (25%)	59 (24%)	
Unknown	101 (3.6%)	87 (3.4%)	14 (5.7%)	

* Statistically significant *p*‐value < 0.05.

**Figure 1:**
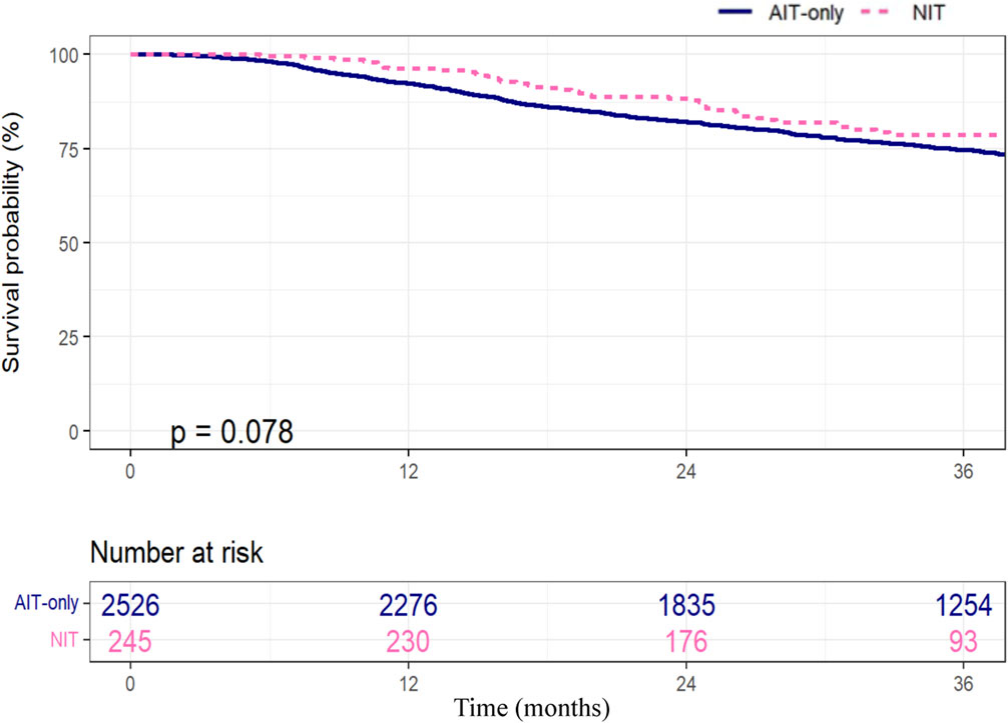
Kaplan–Meier analysis comparing 3‐year overall survival between patients with clinical stage III melanoma who received neoadjuvant immunotherapy (NIT, pink‐dashed) and adjuvant‐only immunotherapy (AIT, blue‐solid).

**Figure 2:**
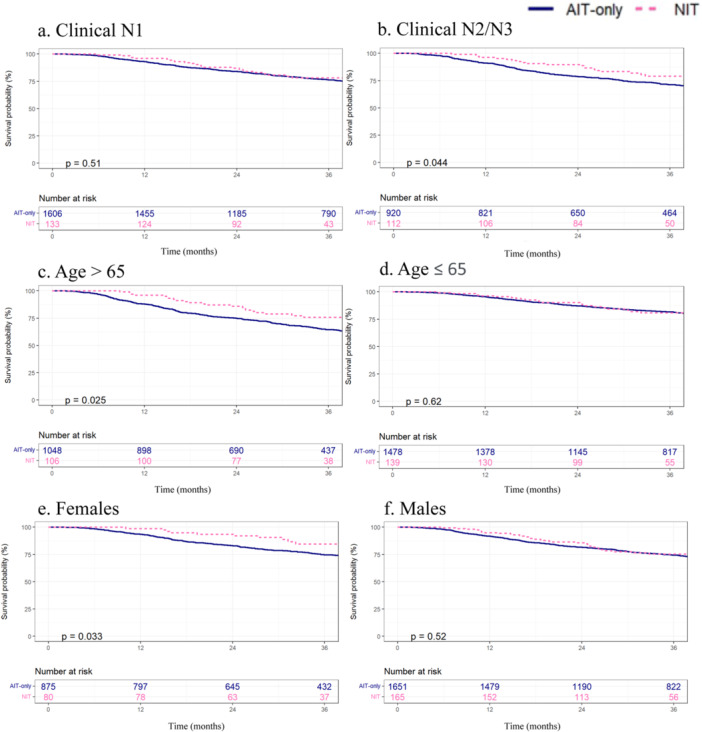
Subgroup Kaplan–Meier analyses comparing 3‐year overall survival on the following groups who received neoadjuvant immunotherapy (NIT, pink‐dashed) and adjuvant‐only immunotherapy (AIT, blue‐solid): (a) Clinical N1, (b) clinical N2/N3, (c) age > 65, (d) age ≤ 65, (e) females, and (f) males.

With a median follow‐up of 44 months, there was no difference in 3‐year OS between the two cohorts on Kaplan–Meier analysis, with OS of 79% (95% CI 73%–85%) for the NIT group and 75% (95% CI 73%–76%) for the AIT‐only group (*p* = 0.078) (Figure 1). When considering only patients clinical N1 disease, there was no difference in 3‐year OS between the NIT (78%, 95% CI 71%–87%) and AIT‐only (76%, 95% CI 74%–79%) groups (Figure 2a). However, among patients presenting with higher clinical N category disease (N2/N3), the NIT group demonstrated a significantly better OS of 79% (95% CI 71%–88%) compared to 71% (95% CI 68%–75%) for the AIT‐only group (*p* = 0.044) (Figure 2b). This survival advantage persisted on Cox multivariable analysis with a hazard ratio (HR) of death of 0.61 for the NIT group compared to AIT‐only group (95% CI 0.38–0.97, *p* = 0.037). This model was adjusted for factors found to be significantly associated with survival in a series of univariable analyses, including patient age, CDCC score, tumor ulceration, and N category (Table 2). Notably, Breslow thickness was not included in our multivariable model as it was not associated with survival on univariable analysis.

**Table 2:** Univariable Cox analyses examining the relationship between patient‐, disease‐, and hospital‐related factors and 3‐year survival.

	HR	95% CI	*p*‐value
Age	1.03	1.03–1.04	< 0.001*
Sex			
Female (baseline)			
Male	1.11	0.96–1.29	0.17
Race			
White (baseline)			
Black	0.70	0.25–1.99	0.50
AAPI	1.08	0.61–1.92	0.78
Other/unknown	1.39	0.52–3.72	0.51
CDCC			
0 (baseline)			
1	1.27	1.04–1.55	0.017*
2	1.49	1.06–2.08	0.021*
3+	3.00	2.24–4.01	< 0.001*
N category			
1 (baseline)			
2	1.10	0.94–1.29	0.24
3	1.43	1.16–1.76	0.001*
Ulceration	1.82	1.56–2.14	< 0.001*
Breslow depth (mm)	1.01	1.00–1.02	0.21
Primary site			
Extremity (baseline)			
Head/neck	0.85	0.71–1.02	0.075
Trunk	1.00	0.85–1.18	0.98
Hospital status			
Nonacademic (baseline)			
Academic	0.85	0.74–0.98	0.022*
Immunotherapy			
Adjuvant (baseline)			
Neoadjuvant	0.65	0.42–0.99	0.045*

* Statistically significant *p*‐value < 0.05.

On subgroup analysis by age, patients older than 65 who received NIT had a significantly better 3‐year OS of 76% (95% CI 67%–85%) compared to 65% (95% CI 61%–68%) for AIT‐only (*p* = 0.025) (Figure 2c). The significance of this finding did not persist on Cox regression (HR 0.66, 95% CI 0.44–1.00, *p* = 0.051). Patients aged 65 and younger did not have a significant difference in OS between the NIT (81%, 95% CI 74%–89%) and AIT‐only (82%, 95% CI 80%–84%) cohorts (Figure 2d). On subgroup analysis by sex, female NIT patients had a significantly better 3‐year OS of 85% (95% CI 76%–94%) compared to female AIT‐only patients with an OS of 75% (95% CI 72%–78%; *p* = 0.033) (Figure 2e). This finding remained significant in the multivariable analysis (HR 0.53, 95% CI 0.29–0.98, *p* = 0.042). Male patients had no 3‐year OS difference between NIT (75%, 95% CI 68%–83%) and AIT‐only groups (75%, 95% CI 72%–77%) (Figure 2f).

## 4 Discussion

Recent work has focused on the application of immunotherapy for melanoma in the neoadjuvant setting, particularly within the clinical trial setting [14, 15, 17, 18, 20, 21]. Less is known about the impact of these therapies in broader practice. In our study, we found that between 2016 and 2020, likely when many, if not most patients, were being treated with NIT in clinical trials, patients receiving NIT were more likely to have more advanced nodal disease (N2/N3) as compared to those receiving AIT‐only.

Despite the difference in N category between the NIT and AIT‐only groups, there was no difference in 3‐year OS. On subgroup analyses, however, we found that NIT was associated with improved survival amongst N2/N3 patients, female patients, and patients older than 65, though the significance of the survival difference in the older cohort did not persist on multivariable analysis. Differential benefit by age and sex was also observed in the SWOG 1801 trial, though interestingly, investigators found that male patients saw greater benefit with NIT than female patients.

While we have excellent clinical trial data demonstrating the advantage of NIT in mitigating recurrence risk, there is still a paucity of evidence showing a survival benefit. In addition, we know that real‐world circumstances do not always mimic the idealized clinical trial setting, and so identifying a signal for the efficacy of NIT using observational data from a large, heterogenous national cohort, including from various clinical trial settings with variable treatment durations and regimens, offers new insight.

This study faces several limitations, including the inability to determine which patients participated in clinical trials, limiting the generalizability. We do not have information about the specific type and duration of immunotherapy received (including dual checkpoint blockade). However, we do know that the median time to surgery for the NIT group was 127 days, or approximately 4 months, compared to 33 days for the AIT‐only group. This is compatible with patients receiving a median number of 3 (or fewer) doses of NIT, a duration of which has shown efficacy in multiple NIT trials, including the SWOG 1801 trial. We are also missing information pertaining to pathological response in the NIT group, as well as patterns and timing of recurrence and subsequent treatments. There were likely patients excluded from our study who were planned for AIT‐only but who experienced early postoperative recurrences and never received immunotherapy. This introduces selection bias into our study and likely disproportionately enriches our AIT‐only population with patients with more favorable disease biology. If so, we may be underestimating the true survival advantage associated with NIT. In addition, since recurrence data is not available, some patients initially classified as stage III who quickly progressed after surgery and received immunotherapy treatment may have been included in the “adjuvant” immunotherapy arm. While a limitation, this would be more consistent with an intention to treat analysis comparing AIT‐only to NIT. Moreover, data on the BRAF mutational status is lacking, making it uncertain whether there is an imbalance in treatment options between the NIT and AIT‐only groups. By its nature, this retrospective study is also limited by unmeasured confounding, though the baseline characteristics were well balanced across treatment groups apart from N category, for which we performed a subgroup analysis, as well as academic status of the treating hospital, which we adjusted for in our multivariable analyses. Despite limitations, these national data support the continued study of promising NIT approaches, and further prospective long‐term data comparing sequencing of immunotherapy are eagerly awaited to better delineate the potential benefits of NIT in resectable metastatic melanoma.

## 5 Conclusions

While recent clinical trials have shown the efficacy of neoadjuvant immunotherapy in melanoma with regard to recurrence risk, there remains limited evidence regarding survival benefit. Through our analysis, we have found that NIT is given more selectively to clinical stage III patients with more advanced N category disease. Despite significant differences in N category between groups, with more advanced disease noted in the NIT group, there was no difference in OS observed at 3 years. Furthermore, in subgroup analysis, NIT was associated with a survival advantage among N2/N3 patients. These national data support the continued usage and study of NIT approaches in patients with high‐risk resectable melanoma.

## Conflicts of Interest

Giorgos Karakousis—Scientific advisory board participation: Bristol‐Myers‐Squibb, Merck; research institutional funds. Tara Mitchell—Scientific advisory board participation: Bristol‐Myers‐Squibb‐Advisory Board, Merck, Pfizer. The other authors declare no conflicts of interest.

## Synopsis

In this national cohort study comparing survival outcomes for patients with clinical stage III melanoma who underwent either neoadjuvant immunotherapy or immunotherapy only after surgery, the neoadjuvant group had similar survival rates despite presenting with greater nodal disease burden. For some subgroups, neoadjuvant therapy was associated with a survival advantage.

## Data Availability

The data that supports the final findings of this study are available from the corresponding author upon reasonable request.
